# Oviposition Competition Between *Zeugodacus cucurbitae* and *Bactrocera dorsalis* Adults in Five Hosts

**DOI:** 10.3390/insects16040419

**Published:** 2025-04-15

**Authors:** Hongai Su, Jin Zhao, Haikuo Yu, Saleem Jaffar, Zhenyu Hao, Guangwen Liang, Ling Zeng, Yongyue Lu

**Affiliations:** Department of Entomology, South China Agricultural University, Guangzhou 510642, China; hongaisu@163.com (H.S.); zj@chuangheip.com (J.Z.); zengling@scau.edu.cn (L.Z.)

**Keywords:** *Zeugodacus cucurbitae*, *Bactrocera dorsalis*, oviposition, competition, host

## Abstract

The *Zeugodacus cucurbitae* and *Bactrocera dorsalis* are economically significant pests that share similar habitats and host plants, leading to intense interspecific competition. To elucidate their competitive interactions, this study investigated the oviposition competition behavior of mixed laboratory populations on five common host plants: pumpkin (*Cucurbita moschata*), cucumber (*Cucumis sativus*), winter melon (*Benincasa hispida*), bitter melon (*Momordica charantia*) and guava (*Psidium guajava*). The results exhibited that among the five hosts tested, *B. dorsalis* exhibited a significant competitive advantage in oviposition on pumpkin and bitter melon, with its fecundity greater than that of *Z. cucurbitae* across all ratios. Conversely, *Z. cucurbitae* showed a clear preference for cucumber. Except at the extreme ratio of 16:4, where *B. dorsalis* laid slightly more eggs than that of *Z. cucurbitae*, *Z. cucurbitae* laid more eggs at all ratios. The results can provide a reference for further investigation on the oviposition selectivity and interspecific competition between the adults of *Z. cucurbitae* and *B. dorsalis*.

## Simple Summary

As significant invasive pests, the melon fruit fly (*Zeugodacus cucurbitae*) and the oriental fruit fly (*Bactrocera dorsalis*) cause significant economic losses in agricultural and forestry production. Their high reproductive capacity and adaptability further complicate control efforts. This paper investigates the oviposition competition between laboratory populations of these two fruit flies on five commonly infested hosts: pumpkin (*Cucurbita moschata*), cucumber (*Cucumis sativus*), winter melon (*Benincasa hispida*), bitter melon (*Momordica charantia*) and guava (*Psidium guajava*). The oviposition competition between *B. dorsalis* and *Z. cucurbitae* on different hosts shows significant differences. *B. dorsalis* exhibits a competitive advantage on hosts such as pumpkin and winter melon, while *Z. cucurbitae* performs better on cucumber. In mixed groups, the oviposition capabilities of the two species influence each other. This research lays a foundation for understanding the population dynamics and developmental succession of these two fruit fly species while also contributing to the development of effective prevention and control strategies.

## 1. Introduction

The melon fruit fly, *Zeugodacus cucurbitae*, and the oriental fruit fly, *Bactrocera dorsalis*, are significant quarantine pests belonging to Diptera, family Tephritidae. These species are primarily distributed across tropical, subtropical and warm temperate regions, with a notable presence in Guangdong, Yunnan and Fujian provinces of Southern China [1,2,3]. Both of them share similar phenotypical appearances, possess high reproductive capacities and exhibit broad host ranges. *Z. cucurbitae* can infest over 125 species, while *B. dorsalis* affects more than 250 species, encompassing a diverse array of fruits, vegetables and florae [3,4,5]. Common hosts include cucumber, squash, bitter melon, pumpkin and melon [5,6,7,8]. The adult flies lay their eggs within the flesh of fruits and vegetables, and the hatched larvae feed on the surrounding tissue [9,10]. Additionally, these two species exhibit similar behavioral patterns and comparable damage profiles. Consequently, there exists a competitive relationship between these two fruit fly species regarding oviposition on shared host plants. According to loss statistics, the annual economic damage caused by *B. dorsalis*, *Z. cucurbitae* and *Bactrocera tau* in Guangdong province is estimated to range between 33.67 billion and 129.87 billion yuan, with control costs reaching as high as 684 million yuan [11].

Interspecific competition is a widespread ecological phenomenon that occurs when two or more species interact due to their shared reliance on limited resources [12]. Within the Tephritidae family, numerous studies have highlighted the significance of interspecific competition and competitive displacement among various species. However, the complete exclusion of one species by another is rarely observed [13,14,15,16,17,18]. For instance, both *Z. cucurbitae* and *B. tau* exhibited strong competitiveness on their preferred hosts. At high density (8:8), *B. tau* demonstrated a greater competitive advantage over *Z. cucurbitae* [19]. However, this competitive disparity was less pronounced at lower densities (1:1, 2:2 and 4:4) [18,20]. Furthermore, intraspecific competition between these two species of fruit flies significantly influences their growth and development [21]. In *B. dorsalis* and *Bactrocera correcta*, it was found that there was a clear advantage on the side of larger population proportional levels [21]. Similar competitive relationships have been documented in other insect species. For instance, during interspecific competition between *Coccinella septempunctata* and *Harmonia axyridis*, *H. axyridis* exhibited relative dominance [22]. Likewise, in the competition between *Frankliniella occidentalis* and *Thrips tabaci*, the experimental populations of *F. occidentalis* rapidly replaced the population of *T. tabaci*, demonstrating a significant inhibitory effect on the latter’s reproduction [23]. These results suggest that the complex interspecific competition among insects occurs at all stages of the insect life cycle and is influenced by the relative intensity of intraspecific competition [24]. As a consequence, comparative studies of fruit flies can enhance our understanding of the interaction mechanisms between competing species and can improve predictive capabilities and management strategies for fruit flies. While similar studies have been conducted, research addressing oviposition competition between adult *Z. cucurbitae* and *B. dorsalis* remains scarce.

Our study aimed to compare the oviposition preferences of *Z. cucurbitae* and *B. dorsalis* on five hosts by comparing interspecific and intraspecific interactions. By measuring the egg-laying rates of these two fly species at varying densities, we explored the oviposition competition patterns of adults under different density conditions. This research provides valuable insights into the interspecific relationships between *Z. cucurbitae* and *B. dorsalis*, as well as contributing to the integrated management of Tephritidae pests.

## 2. Materials and Methods

### 2.1. Insect Rearing and Test Host

*Z. cucurbitae* and *B. dorsalis* larvae were collected from bitter melon fields at the teaching experiment site of South China Agricultural University (latitude 23.1598° N, longitude 113.3449° E). The larvae were maintained on *Cucurbita pepo* before being transferred to small plastic boxes filled with sand for pupation. The pupae were kept at a temperature of 27 °C ± 1 °C and a relative humidity of 75% ± 1% until the adults emerged, which were then provided with an artificial diet of yeast extract and dry sugar mixed in a 1:1 ratio (*w*/*w*) [25], and housed in wooden cages measuring 35 cm × 35 cm × 35 cm [26]. Additionally, pumpkin was made available to the adults for oviposition. The experimental hosts included five species: pumpkin (*Cucurbita moschata*), cucumber (*Cucumis sativus*), winter melon (*Benincasa hispida*), bitter melon (*Momordica charantia*) and guava (*Psidium guajava*).

### 2.2. Determination of the Optimal Egg-Laying Time for Fruit Flies

After mating, one female adult of *B. dorsalis* or one female adult of *Z. cucurbitae* was placed in a separate insect cage measuring 35 cm × 35 cm × 35 cm, each supplied with artificial diet and water. A pumpkin piece approximately 4 cm × 4 cm × 1 cm in size was placed in each cage to facilitate oviposition by the female adults. The oviposition behavior on the pumpkin slices was continuously monitored, and a corresponding diagram of the slice was sketched in a paper. Each instance of a female inserting its ovipositor into the slice was marked at the precise location on the diagram. From 08:00 to 17:00, the number of oviposition holes made by the fruit flies was recorded continuously. To eliminate the potential influence of experimental factors such as light, temperature and humidity, all insect cages were maintained in a climate-controlled rearing room with stable environmental conditions throughout the experiment. The rearing room was precisely regulated at a constant temperature of 27 ± 1 °C and a relative humidity of 75 ± 1%. Additionally, artificial lighting (500 lux) was used to ensure the precise control of photoperiod conditions. This standardized setup guaranteed the consistency and stability of the experimental environment. The experiment was repeated five times.

### 2.3. Analysis of the Number of Oviposition Holes and Eggs

Ten female adults of *B. dorsalis* and *Z. cucurbitae* were placed in separate insect cages, each containing a pumpkin piece measuring approximately 4 cm × 4 cm × 1 cm. To minimize the interference with the oviposition behavior of female flies as much as possible, during the experiment, we closely observed the oviposition actions of female flies. Once a female fly was observed inserting its ovipositor into a pumpkin slice, a transparent plastic cup was immediately gently inverted to cover the slice. After the female fly completed oviposition and left the pumpkin piece, it was removed from the cage, and the number of oviposition holes on it was recorded. For individuals that were obviously disturbed, their relevant data will be excluded in the subsequent analysis. This experiment was repeated thirty times.

### 2.4. Interspecific Competition Among Different Proportions of Adults on Five Host Species

Mixed groups: mated female *B. dorsalis* and *Z. cucurbitae* were mixed in seven different ratios: 16:4, 14:6, 12:8, 10:10, 8:12, 6:14 and 4:16 pairs. Then, the fruits of the above five host species were cut into small pieces of 4 cm × 4 cm ×1 cm and placed in the cages containing the above females in different ratios. Single population: 16, 14, 12, 10, eight, six and four pairs of adult female *B. dorsalis* or *Z. cucurbitae* were used as single groups for control experiments. The remaining experimental methods were identical to those used for the mixed groups. The experiment was conducted daily from 9:00 to 13:00, lasting for a total of 4 h. By monitoring the positions where the ovipositors of the two species were inserted into the pumpkin slices in real time and marking the corresponding positions on recording paper, we accurately distinguished the number of oviposition holes made by *B. dorsalis* and *Z. cucurbitae*. The number of oviposition holes in each treatment was recorded, and their competition patterns were analyzed. Each treatment was repeated five times.

### 2.5. Statistical Analyses

Statistical analyses of data were performed using Prism 8.0 (GraphPad Software, Boston, MA, USA). We performed the *t*-test for unpaired comparisons between two groups of data. Differences were considered significant at *p* < 0.05. Values are reported as mean ± standard error of the mean (SEM).

## 3. Results

### 3.1. The Optimal Egg-Laying Time for B. dorsalis and Z. cucurbitae

**Figure 1:**
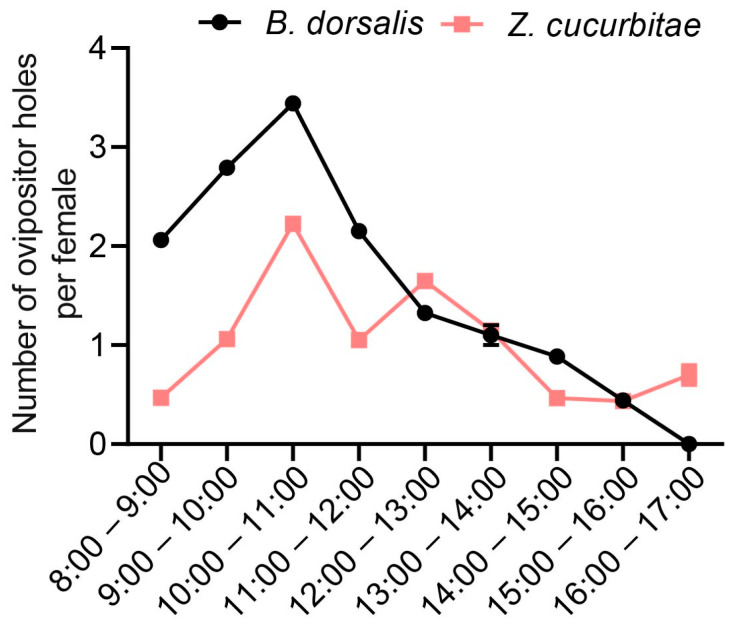
The number of oviposition holes made by individual *Z. cucurbitae* and *B. dorsalis* at different time intervals.

To determine the peak oviposition periods of the two fruit fly species, we conducted a statistical analysis of their oviposition sites at different time intervals. Observations were made between 9:00 and 17:00. The results indicated that the optimal oviposition times for *B. dorsalis* were 9:00–10:00 and 10:00–11:00 (Figure 1). For *Z. cucurbitae*, the peak times were 10:00–11:00 and 12:00–13:00 (Figure 1). Based on these findings, we selected a daily time frame from 9:00 to 13:00 for conducting oviposition competition experiments, covering a total of 4 h.

### 3.2. Analysis of the Number of Ovipositor Holes and the Number of Eggs Laid by Adults for B. dorsalis and Z. cucurbitae

**Figure 2:**
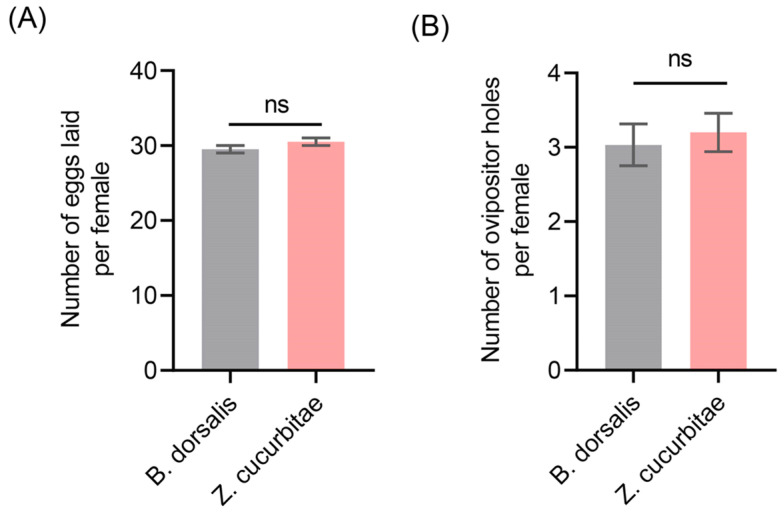
Analysis of oviposition behavior in *Z. cucurbitae* and *B. dorsalis*. (**A**) Number of oviposition holes made by individual females of *Z. cucurbitae* and *B. dorsalis*; (**B**) Number of eggs laid by individual females of *Z. cucurbitae* and *B. dorsalis*. We conducted an unpaired *t*-test to compare the two groups of data. The standard error is represented by the error bar. The ns indicates no significant difference.

To investigate whether differences existed between the number of oviposition holes and the number of eggs laid, we conducted a statistical analysis on the *B. dorsalis* and *Z. cucurbitae*. The results indicated that both species had an average of approximately 30 eggs per female oviposition holes, with no significant differences observed between them (Figure 2A). Further analysis revealed that the average number of oviposition holes by a single female *Z. cucurbitae* was 3.03 while, for *B. dorsalis*, it was 3.13, again showing no significant difference (Figure 2B). For statistical simplicity, based on these findings, we used the number of oviposition holes as a representative measure of egg number for both species in subsequent oviposition competition experiments.

### 3.3. Competition Analysis of Two Species Adults on Five Hosts in Mixed Groups

**Figure 3:**
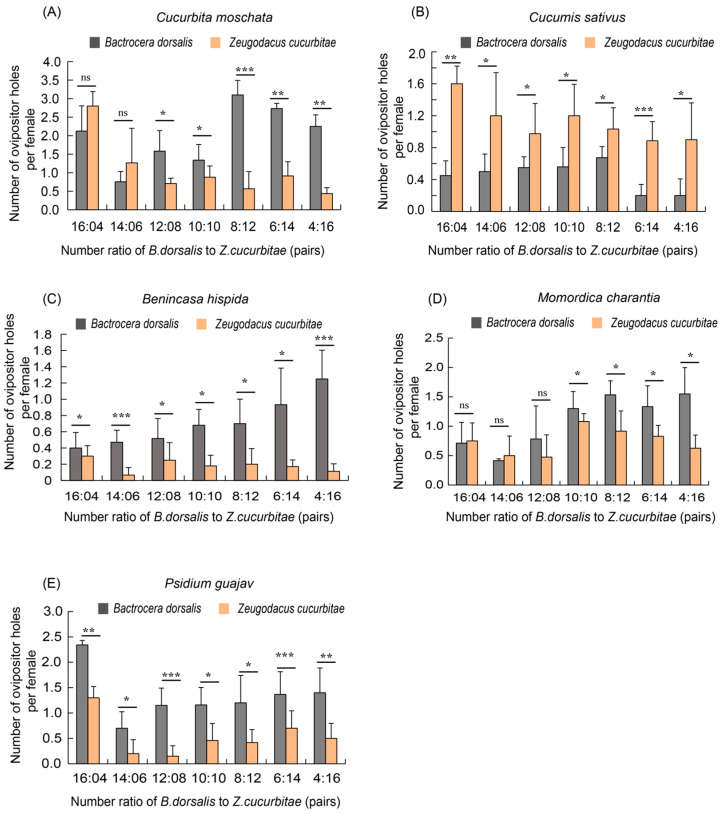
A comparison of the number of oviposition holes made by *Z. cucurbitae* and *B. dorsalis* in mixed groups on five hosts. (**A**) Number of ovipositor holes on pumpkin (*C. moschata*) by mixed groups; (**B**) Number of ovipositor holes on cucumber (*C. sativus*) by mixed groups; (**C**) Number of ovipositor holes on winter melon (*B. hispida*) by mixed groups; (**D**) Number of ovipositor holes on bitter melon (*M. charantia*) by mixed groups; (**E**) Number of ovipositor holes on guava (*P. guajava*) by mixed groups. The standard error is represented by the error bar. The ns indicates no significant difference, while an asterisk indicates values significantly different from the control value determined from unpaired *t*-test (* *p* < 0.05, ** *p* < 0.01, *** *p* < 0.001).

The results of the competition experiments revealed significant differences in the oviposition behavior of the two fruit fly species across five host plants. On the pumpkin and bitter melon hosts, when the numbers of the two species were equal or when *B. cucurbitae* was more numerous (10:10, 8:12, 6:14, and 4:16), the number of oviposition holes made by *B. dorsalis* was significantly higher than that of *B. cucurbitae*. However, when *B. dorsalis* was more numerous (16:4 and 14:6), there was no significant difference in oviposition holes between these two species (Figure 3A). On the winter melon and guava hosts, the number of oviposition holes produced by *B. dorsalis* was still dominant across all seven ratios (Figure 3C,E). In contrast, on the cucumber host, the number of oviposition holes made by *Z. cucurbitae* was significantly higher than that of *B. dorsalis* (Figure 3B).

Specifically, for *B. dorsalis*, the highest number of oviposition holes was observed on pumpkin with eight pairs of females, averaging 3.1 (Figure 3A). Conversely, the lowest number of oviposition holes was observed on cucumber with six to four pairs of females, averaging 0.4 (Figure 3B). For *Z. cucurbitae*, on cucumber as a host with four females, the highest number of oviposition holes was 1.6 (Figure 3B), while on winter melon with six pairs of females, the lowest number was only 0.1 (Figure 3C).

In summary, in the mixed groups, except for the cucumber hosts, the competitive ability of *B. dorsalis* was significantly greater than that of *Z. cucurbitae* under conditions of medium to low density on the pumpkin, bitter melon, winter melon and guava hosts.

### 3.4. Competition Analysis of B. dorsalis Adults on Five Hosts in Single and Mixed Groups

**Figure 4:**
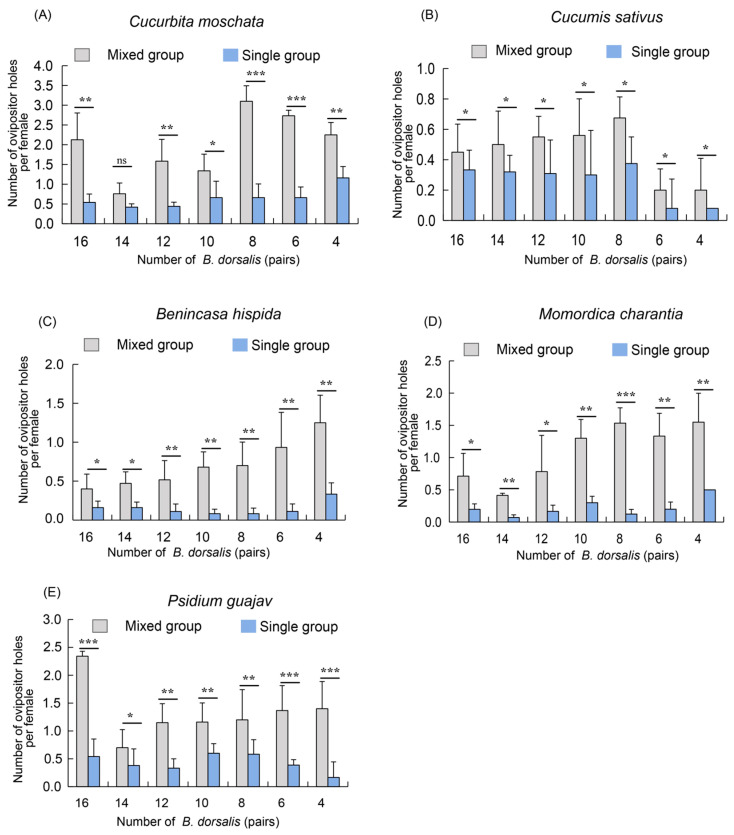
A competition analysis of adult *B. dorsalis* on five hosts in single and mixed groups. (**A**) Number of ovipositor holes of adult *B. dorsalis* on pumpkin (*C. moschata*) in single and mixed groups; (**B**) Number of ovipositor holes of adult *B. dorsalis* on cucumber (*C. sativus*) in single and mixed groups; (**C**) Number of ovipositor holes of adult *B. dorsalis* on winter melon (*B. hispida*) in single and mixed groups; (**D**) Number of ovipositor holes of adult *B. dorsalis* on bitter melon (*M. charantia*) in single and mixed groups; (**E**) Number of ovipositor holes of adult *B. dorsalis* on guava (*P. guajava*) in single and mixed groups. The standard error is represented by the error bar. The ns indicates no significant difference, while an asterisk indicates values significantly different from the control value determined from unpaired *t*-test (* *p* < 0.05, ** *p* < 0.01, *** *p* < 0.001).

To further evaluate the competitive ability of *B. dorsalis* in mixed and single groups, we used corresponding numbers of single *B. dorsalis* groups as controls for comparison. On five host plants, the number of oviposition holes formed by *B. dorsalis* was significantly higher in the mixed groups than in the corresponding single groups across all seven ratios (Figure 4A–E). Specifically, on the pumpkin, cucumber and bitter melon hosts, the highest number of oviposition holes was observed with eight pairs of females, while the lowest was recorded with fourteen pairs (Figure 4A,B,D). Additionally, on the cucumber and winter melon hosts, the number of oviposition holes in the mixed groups increased gradually as the number of female pairs decreased, compared to the single groups (Figure 4B,C).

In summary, these results indicate that *B. dorsalis* exhibits significantly stronger competitive abilities in mixed groups compared to single groups under identical individual densities.

### 3.5. Competition Analysis of Z. cucurbitae Adults on Five Hosts in Single and Mixed Groups

**Figure 5:**
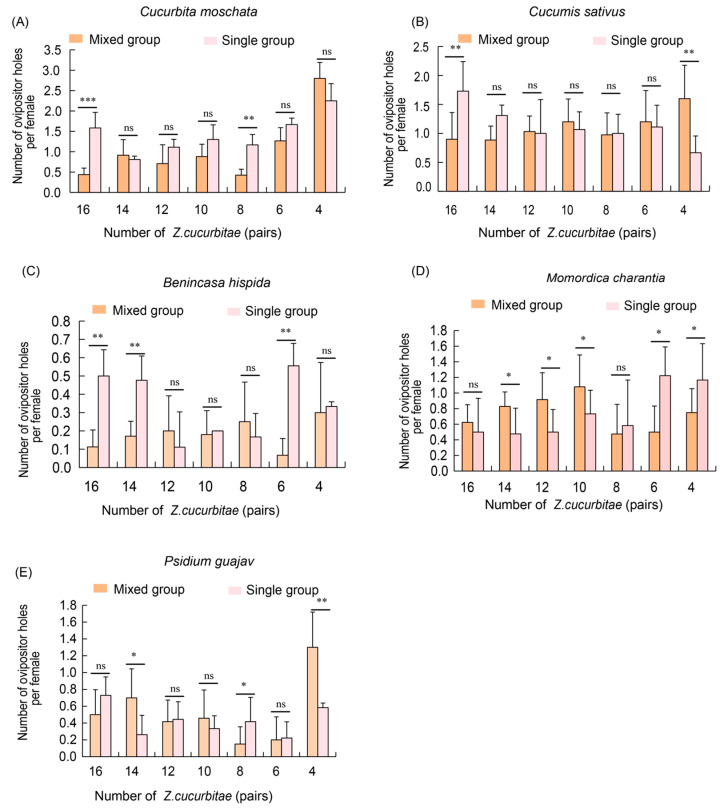
Competition analysis of adult *Z. cucurbitae* on five hosts in single and mixed groups. (**A**) Number of ovipositor holes of adult *Z. cucurbitae* on pumpkin (*C. moschata*) in single and mixed groups; (**B**) Number of ovipositor holes of adult *Z. cucurbitae* on cucumber (*C. sativus*) in single and mixed groups (**C**) Number of ovipositor holes of adult *Z. cucurbitae* on winter melon (*B. hispida*) in single and mixed groups; (**D**) Number of ovipositor holes of adult *Z. cucurbitae* on bitter melon (*M. charantia*) in single and mixed groups; (**E**) Number of ovipositor holes of adult *Z. cucurbitae* on guava (*P. guajava*) in single and mixed groups. The standard error is represented by the error bar. The ns indicates no significant difference, while an asterisk indicates values significantly different from the control value determined from unpaired *t*-test (* *p* < 0.05, ** *p* < 0.01, *** *p* < 0.001).

Similarly, we evaluated the competitive ability of *Z. cucurbitae* in mixed and single groups. The results showed that on pumpkin hosts, the number of oviposition holes in the single population was significantly higher compared to the mixed groups at densities of 16 pairs and eight pairs, while no significant differences were observed at other ratios (Figure 5A). On cucumber hosts, at a density of 16 pairs, the single population had a significantly greater number of oviposition holes compared to the mixed groups. However, at a density of four pairs, the mixed groups showed significantly more oviposition holes than the single groups (Figure 5B). No significant differences were found between the two groups at other ratios (Figure 5B). For winter melon hosts, in the mixed groups, the number of oviposition holes was significantly higher than that in the single groups at densities of 16 pairs, 14 pairs and six pairs (Figure 5C). In bitter melon, the mixed groups had a significantly greater number of oviposition holes than the single groups at densities of 14 pairs, 12 pairs and 10 pairs. Conversely, at densities of six pairs and four pairs, the single groups exhibited significantly more oviposition holes than the mixed groups. No significant differences were observed between the groups at densities of 16 pairs and eight pairs (Figure 5D). Finally, on guava hosts, the mixed groups exhibited a significantly higher number of oviposition holes than the single population at densities of 14 pairs and four pairs. However, at densities of 16 pairs and eight pairs, the single groups had significantly more oviposition holes than the mixed groups. No significant differences were observed at densities of 12 pairs, one pair or six pairs (Figure 5E).

In summary, on some host plants, *Z. cucurbitae* single groups showed significant competitive advantages at higher densities (16 and eight pairs), while in other cases (four pairs), the mixed groups showed a stronger competitive ability. This suggests that host plant type and population structure have a significant effect on the competitive ability of *Z. cucurbitae*.

## 4. Discussion

The research found that *Z*. *cucurbitae* and *B*. *dorsalis* preferred ovipositing on pumpkin and cucumber, respectively, while showing minimal interest in bitter melon. This indicates that the oviposition competition between the two species is host-specific. A similar phenomenon has also been observed between *B*. *tau* and *Z.cucurbitae* [18]. Previous studies have shown that *B. dorsalis* lays the most eggs on its preferred varieties, resulting in higher survival rates [27]. Liu et al. investigated the egg-laying preferences of two species of fruit flies, *B. dorsalis* and *B. correcta*, across six host plants. They found that *B. dorsalis* preferred guava, sweet orange and banana while *B. correcta* favored guava and sweet orange. Notably, neither species showed a preference for papaya or tomato [28]. In addition to the suitability of the host for egg laying, Craig et al. suggested that host selection is closely related to the host’s ability to promote the growth and development of the offspring [29]. This phenomenon reflects the long-term interactions between insects and their hosts, leading to egg-laying choices that enhance the reproduction and development of their own populations. Host preference is influenced by various physical factors, including color, shape, size and morphology [30,31], as well as the physiological conditions of the flies [32,33]. In addition, environmental factors such as temperature, light and humidity have a significant impact on the growth, development and reproduction of fruit flies. Studies have shown that, under the condition of 17–21 °C, the life cycle of *B*. *dorsalis* is longer and the survival rate is lower while, under the condition of 25–29 °C, the population growth index is higher, indicating that this temperature range is suitable for the development and reproduction of *B*. *dorsalis* [34]. Light intensity is also one of the key factors affecting the reproductive behavior of fruit flies. Adult fruit flies usually initiate mating behavior under low light intensity (<1000 lux). However, when the light intensity exceeds 20,000 lux, their reproductive behavior will be significantly affected [35]. In this study, in order to minimize the interference of external factors on the oviposition of fruit flies, the experiment was conducted under the conditions of constant temperature and humidity and fixed light source (27 °C ± 1 °C, 75% ± 1%, 500 lux). Therefore, the oviposition behavior of these two fruit fly species is determined by a combination of multiple factors.

In this study, we employed a transparent plastic cup covering method to minimize interference with the oviposition behavior of female fruit flies, thereby assessing the relationship between the number of eggs laid by fruit flies and the number of oviposition holes. However, during the experiment, we found that placing the transparent cup might touch other female flies, which can easily disturb the females that are in the process of oviposition. Moreover, the presence of the transparent cup could potentially alter the oviposition site selection of female flies, thereby affecting factors such as oviposition frequency and mating time. To avoid the above situation, we operated as gently as possible when placing the transparent cup, completed the operation process as quickly as possible and eliminated the data that were affected. Overall, although the transparent cup covering may have some interference with the oviposition behavior of female flies, this interference is not significant in this study. Future research still needs to explore new methods to further reduce interference with the oviposition behavior of female flies. Our results indicate the role of both intraspecific and interspecific competition between the two fruit fly species. *B. dorsalis* exhibited significantly stronger competitive abilities than *Z. cucurbitae* on most host plants, and its competitive ability in mixed groups was also superior to that in single groups. This suggests that interspecific competition has a significant impact on the oviposition of *B. dorsalis*, resulting in a “disruption-weakening” phenomenon. This may be related to the disturbance resistance of *Z. cucurbitae and B. dorsalis*. Observations revealed that *Z. cucurbitae* has a relatively weak disturbance response: when disturbed while crawling or laying eggs on a host, it immediately flies away. In contrast, *B. dorsalis* is less easily startled and exhibits stronger resistance to disturbance. This aligns with the results of previous oviposition competition studies between *Sitophilus zeamais*, *Rhizopertha dominica* [36] and *Z. cucurbitae* and *B. tau* [20,21]. In certain host plants, the presence of one pest in a mixed group can influence the oviposition capacity of another pest, either promoting or inhibiting its oviposition behavior. Additionally, population density is a crucial factor in determining the intensity of competition. Previous studies have shown that higher densities lead to scarcer resources, resulting in more intense competition [29]. In artificial diets, competition between *Ceratitis capitata* and *B. dorsalis* is not intense and occurs only at very high population densities [37].

This study simulated the oviposition competition among adult fruit flies at different densities, filling in gaps in the research on interspecific competition between *Z. cucurbitae* and *B. dorsalis*. However, numerous field factors influence interspecific competition between invasive species and native populations [12,38], including natural enemies, climate conditions and insecticide resistance [39], all of which can significantly impact interspecific competition [40,41]. For instance, the presence of natural enemy wasps *Fopius arisanus* (egg) and *Diachasmimorpha longicaudata* (larvae), which are natural parasitoids of the fruit fly, could alter the competitive outcomes by preferentially targeting one species over the other. Additionally, the development of insecticide resistance in one species could shift the competitive balance, allowing it to dominate in agricultural settings where chemical control measures are frequently applied.

Furthermore, other ecological interactions, such as resource availability, host plant preferences and microbial symbionts gut bacteria (e.g., *Enterobacter*, *Klebsiella*, *Pseudomonas*), may also play a role in shaping the competitive dynamics between *Z. cucurbitae* and *B. dorsalis*. For example, if one species has a broader host range or can utilize a wider variety of fruits for oviposition, it may outcompete the other in diverse agricultural landscapes. Similarly, symbiotic bacteria that enhance nutrient acquisition or detoxify plant defenses could provide a competitive edge to one species.

Therefore, further research is necessary to understand the dynamics and mechanisms of competition between *Z. cucurbitae* and *B. dorsalis* in field groups. Field-based studies could include monitoring the distribution and abundance of both species in different agroecological zones, assessing the impact of natural enemies under varying environmental conditions and evaluating the role of insecticide resistance in competitive outcomes. Additionally, experimental field trials that manipulate factors such as host plant diversity, pesticide application regimes and the presence of natural enemies could provide deeper insights into the complex interactions governing interspecific competition. Such research would not only enhance our understanding of the ecological and evolutionary processes driving competition but also inform more effective IPM strategies to mitigate the impacts of these invasive species on agriculture.

## Data Availability

The data presented in this study are available on request from the corresponding author.
